# Meta-Analysis of Atrial Fibrillation Ablation in Patients with Systolic Heart Failure

**DOI:** 10.1155/2019/8181657

**Published:** 2019-01-06

**Authors:** Mohammed Ruzieh, Andrew J. Foy, Nader M. Aboujamous, Morgan K. Moroi, Gerald V. Naccarelli, Mehrdad Ghahramani, Shaffi Kanjwal, Joseph E. Marine, Khalil Kanjwal

**Affiliations:** ^1^Penn State Heart and Vascular Institute, Hershey, PA, USA; ^2^Penn State Department of Internal Medicine, Hershey, PA, USA; ^3^Penn State College of Medicine, Hershey, PA, USA; ^4^St Marys of Michigan, Saginaw, MI, USA; ^5^John's Hopkins University School of Medicine, Baltimore, MD, USA; ^6^Michigan State University, McLaren Greater Lansing, MI, USA

## Abstract

Atrial fibrillation (AF) and heart failure (HF) are two common conditions that often coexist and predispose each to one another. AF increases hospitalization rates and overall mortality in patients with HF. The current available therapeutic options for AF in patients with HF are diverse and guidelines do not provide a clear consensus regarding the best management approach. To determine if catheter ablation for AF is superior to medical therapy alone in patients with coexisting HF, we conducted this systematic review and meta-analysis. The primary outcomes evaluated are left ventricular ejection fraction (LVEF), Minnesota Living with Heart Failure Questionnaire (MLWHFQ) scores, 6-minute walk test (6MWT) distance, heart failure hospitalizations, and mortality. The results are presented as a mean difference for continuous outcome measures and odds ratios for dichotomous outcomes (using Mantel-Haenszel random effects model). 7 full texts met inclusion criteria, including 856 patients. AF catheter ablation was associated with a significant increase in LVEF (mean difference 6.8%; 95% CI: 3.5 – 10.1; P<0.001) and 6MWT (mean difference 29.3; 95% CI: 11.8 – 46.8; P = 0.001), and improvement in MLWHFQ (mean difference -12.1; 95% CI: -20.9 – -3.3; P = 0.007). The risk of all-cause mortality was significantly lower in the AF ablation arm (OR 0.49; 95% CI: 0.31 – 0.77; P = 0.002). In conclusion, atrial fibrillation ablation in patients with systolic heart failure is associated with significant improvement in LVEF, quality of life, 6MWT, and overall mortality.

## 1. Introduction

Atrial fibrillation (AF) and heart failure (HF) are two common conditions that often coexist and can predispose each to one another [1–3]. As the population ages, the prevalence of both conditions is expected to increase. AF increases the risk of stroke, hospitalization from heart failure, length of hospital stay, and overall mortality [4, 5].

The current available therapeutic options for AF in patients with HF are diverse and guidelines do not provide a clear consensus regarding the best approach to management. The strategy of rhythm control using antiarrhythmic drugs (AADs) is not superior to rate control in patients with both AF and HF [6].

In the last decade, several randomized controlled trials (RCTs) have examined the role of catheter ablation in the AF patient population and have demonstrated improvement in left ventricular function and quality of life [2, 3, 7–11].

To determine if catheter ablation for AF is superior to medical therapy alone in patients with coexisting HF, we performed a meta-analysis of the available RCTs.

## 2. Methods

We applied the methods recommended by the Preferred Reporting Items for Systematic Reviews and Meta-Analyses (PRISMA) statement [12].

### 2.1. Data Collection and Extraction

We searched PubMed, Google Scholar, the Cochrane Central Register for RCTs and ClinicalTrials.gov for studies that evaluated AF catheter ablation in patients with HF (latest search date: October 1, 2018). The study protocol was drafted by three of the authors (M.R., M.M., and A.F.) and revised by all coauthors. Two authors (M.R. and M.M.) independently reviewed all articles and abstracts for inclusion. They independently extracted information on sample size, follow-up, and outcomes. Discrepancies were discussed and resolved by consensus.

Key search terms used were atrial fibrillation, catheter ablation, pulmonary venous isolation, heart failure, left ventricular dysfunction, low ejection fraction, functional capacity, quality of life, stroke, hospitalization, mortality, and death. Bibliographies of retrieved studies were hand-searched to identify additional relevant studies.

We included studies that randomized patients with AF and systolic HF to either catheter ablation, medical therapy, or atrioventricular-node ablation with pacemaker implantation.

### 2.2. Outcome and Quality Assessment

The primary outcomes were left ventricular ejection fraction (LVEF), Minnesota Living with Heart Failure Questionnaire (MLWHFQ) scores, 6-minute walk test (6MWT) distance, stroke, heart failure hospitalizations, and mortality. Procedural complications were also summarized.

We used the Cochrane Risk of Bias table and the Grading of Recommendations, Assessment, Development, and Evaluation (GRADE) system, to report risk of bias and quality of study outcomes in each study, respectively.

### 2.3. Statistical Analysis

The primary analyses were performed using RevMan version 5.3 (The Nordic Cochrane Center, The Cochrane Collaboration; Copenhagen, Denmark).

The results are presented as a mean difference for continuous outcome measures (using the inverse variance random effects model) and odds ratios (OR) for dichotomous outcomes (using Mantel-Haenszel random effects model), with 95% confidence interval (CI). We performed sensitivity analyses to ascertain the robustness of the results. We quantified heterogeneity using I^2^, which represents the percentage of variability in the effect risk estimate among studies due to heterogeneity rather than chance (with I^2^ <25% considered as low, I^2^ >75% considered as high, and in between [25% to 75%] as intermediate).

Begg's funnel plots method was used to evaluate for potential publication bias.

A two-sided p-value of <0.05 was considered to be statistically significant.

## 3. Results

### 3.1. Qualitative Synthesis

**Figure 1:**
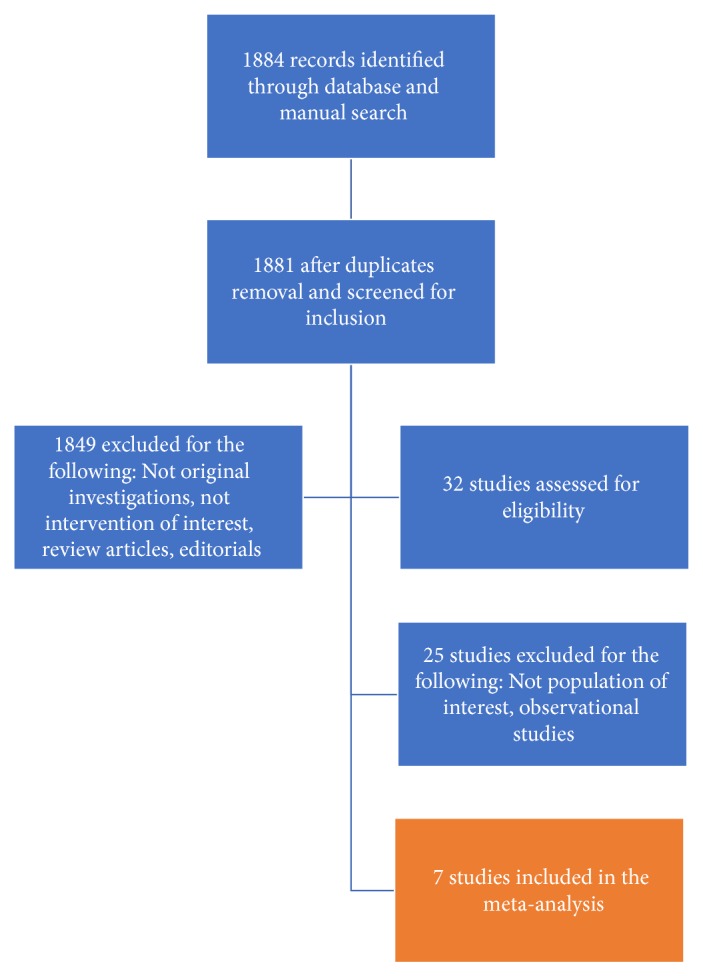
PRISMA diagram showing search strategy results.

Our search identified 1884 studies, of which 7 full texts met inclusion criteria, Figure 1. A total of 856 patients were included (429 patients randomized to catheter ablation and 427 patients randomized to medical therapy alone), with an average age of 63.4 years and a mean follow-up time of 15.2 months. The proportion of males ranged between 73% and 96%. Mean LVEF was 29.9%. The vast majority of patients had persistent AF, and New York Heart Association (NYHA) Functional Classification II-III. Further patient characteristics are listed in Tables 1 and 2.

### 3.2. Risks of Bias and Quality Assessment

For all studies there were limitations in methodology and in outcome assessment (per Cochrane and GRADE criteria), Table 3. Randomization was performed using random number generation in all trials and the baseline characteristics for patients in the ablation arm and the control arm were similar. None of the trials had a sham arm and thus patients were not blinded. Outcome assessment, specifically ejection fraction, was blinded in four trials [2, 3, 8, 9]. Based on the GRADE criteria, we have moderate confidence in the outcome estimates derived from the pooled data.

Crossovers and dropouts were described appropriately in all studies. Crossover occurred in two patients in the study by Jones et. al. [3] and in 46 patients in the CASTLE AF trial [11]. However, intention to treat analysis was performed. Loss to follow-up was largest in the CASTLE AF trial at 33 (9.1%) patients (23 [6.3%] in the ablation arm and 10 [2.8%] in the control arm). Further details are provided in Table 4.

Evaluation of the funnel plots revealed no evidence of publication bias.

### 3.3. Outcomes and Sensitivity Analysis

#### 3.3.1. Arrhythmia Recurrence

**Figure 2:**
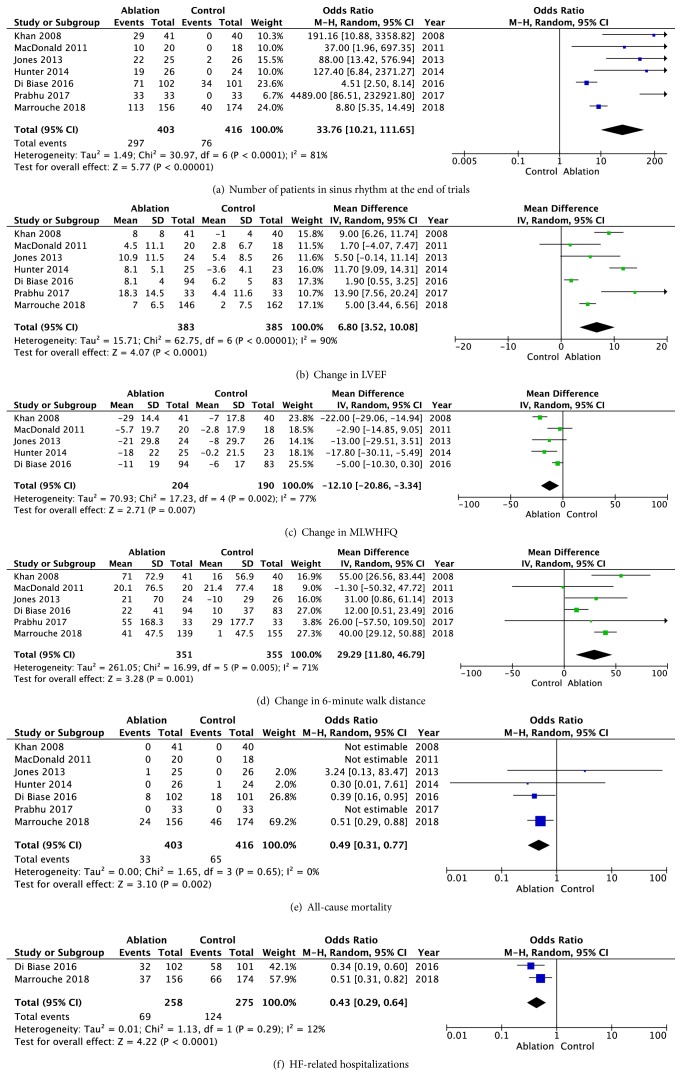
Change in outcomes, all-cause mortality, and HF-related hospitalizations.

AF catheter ablation was an effective therapy and significantly more patients in the AF ablation group were in sinus rhythm at the end of trials (73.7% vs. 18.3%, OR 33.7; 95% CI: 10.2 – 111.7; P< 0.001), Figure 2(a). This difference remained significant when limiting the analysis to the AATAC trial [9] which compared AF ablation to amiodarone, and the CASTLE AF trial [11] which allowed pharmacological rhythm control (71.3% vs. 26.9%, OR 6.4; 95% CI: 3.3 – 12.4; P< 0.001). To achieve this high success rate from AF ablation, repeat intervention was allowed in all trials and the percentage of patients who underwent repeat ablation ranged from 19% to 54%, Table 4.

#### 3.3.2. LVEF

Data for LVEF were available from all included trials. There was significant heterogeneity (I^2^ = 90%). Our confidence in LVEF outcome estimates derived from pooled data is moderate, since four trials [2, 3, 8, 9] assessed LVEF in a blinded fashion.

Compared to medical therapy alone, AF catheter ablation was associated with a significant increase in LVEF (mean difference 6.8%; 95% CI: 3.5 – 10.1; P<0.001), Figure 2(b). One trial compared AF catheter ablation to AV nodal ablation with biventricular pacing as a rate control strategy [7], and even when this trial is excluded, AF catheter ablation was still associated with a significant increase in LVEF (mean difference 6.4%; 95% CI: 2.8 – 10.1; P<0.001). Furthermore, when including only trials that had a blinded assessment of LVEF, AF catheter ablation was still associated with an increase in LVEF, although not statistically significant (mean difference 5.3%; 95% CI: -0.6 – 11.2; P = 0.08).

#### 3.3.3. Quality of Life Based on MLWHFQ Scores

Data on MLWHFQ were available from five trials. There was significant heterogeneity (I^2^ = 77%). Since patients were not blinded to the intervention, our confidence in the MLWHFQ outcome estimate is low.

There was a significant improvement in the MLWHFQ scores in the AF catheter ablation group when compared to the medical therapy group (mean difference -12.1; 95% CI: -20.9 – -3.3; P = 0.007), Figure 2(c). When excluding the trial that compared AF catheter ablation to AV nodal ablation with biventricular pacing as a rate control strategy, heterogeneity became moderate (I^2^ = 33%). However, the mean improvement in the MLWHFQ still favored the AF ablation group (mean difference -8.0; 95% CI: -14.3 – -1.7; P = 0.01).

#### 3.3.4. 6MWT Distance in Meters

Six trials reported 6MWT distance and are included in this analysis. There was significant heterogeneity among these trials (I^2^ = 71%).

The mean increase in 6MWT distance was higher in the AF catheter ablation group compared to the medical therapy group (mean difference 29.3; 95% CI: 11.8 – 46.8; P = 0.001), Figure 2(d). When tested for sensitivity, the removal of any individual trial did not appreciably alter the point estimate or confidence interval in the results.

#### 3.3.5. All-Cause Mortality

Every included trial reported all-cause mortality on follow-up. However, with the exception of the CASTLE AF trial [11], the trials were not designed nor were they powered to detect a mortality difference. Therefore, our confidence in the outcome estimate derived from pooled data is low.

In addition, there was no heterogeneity (I^2^ = 0.00%). The risk of death from any cause was significantly lower in the AF ablation arm (OR 0.49; 95% CI: 0.31 – 0.77; P = 0.002), Figure 2(e). This difference was driven by the CASTLE AF [11] and AATAC trials [9].

Cardiovascular death was only reported by the CASTLE AF trial, and was higher in the medical treatment arm (41 [22.3%] vs. 20 [11.2%]; P = 0.009).

#### 3.3.6. Hospitalizations

HF-related hospitalizations were systematically reported by the AATAC and CASTLE AF trials [9, 11]. Other trials reported procedure related heart failure exacerbations and we summarized this data in the ablation complications section below.

The rare of HF- related hospitalizations was significantly lower in the ablation arm (26.7% vs. 45.1%, OR 0.43; 95% CI: 0.29 – 0.64; P< 0.001), Figure 2(f).

#### 3.3.7. Stroke

This outcome was only systematically reported by the CASTLE AF trial. Other trials reported ablation related stroke and we also summarized this data in the catheter ablation complications section below.

The rate of stroke in the CASTLE AF trial was not significantly lower in the ablation arm compared to the medical treatment arm (5 [3.2%] vs. 11 [6.3%]; P = 0.19).

#### 3.3.8. AF Catheter Ablation Complications

All trials reported complications related to AF catheter ablation. Details of complications and adverse events are listed in Table 5.

There were no procedural related deaths. The overall rate of complications was 33 out of 399 (8.3%), distributed as follows: 2 (0.5%) strokes, 4 (1%) cardiac tamponade, 5 (1.3%) pericardial effusion, 3 (0.8%) pulmonary venous stenosis, 13 (3.3%) access site complications, and 6 (1.5%) heart failure exacerbation.

It would be difficult to compare the rate of adverse events between medical therapy and catheter ablation, as studies allowed patients to remain on rate and/or rhythm control agents post ablation.

## 4. Discussion

This systematic review and meta-analysis of AF ablation in patients with existing heart failure identified seven RCTs. Specifically, one study compared AF ablation to AV nodal ablation with biventricular pacing as a rate control strategy [7], four studies compared AF catheter ablation to rate control using medical therapy [2, 3, 8, 10], one study compared AF ablation to amiodarone [9], and one study compared AF ablation to the combination of rate and rhythm control [11].

Summary evidence from the seven included RCTs showed that AF catheter ablation is associated with a statistically significant increase in LVEF, MLWHFQ, and 6MWT distance, with mean differences of 6.8%, -12.1, and 29.3, respectively. Although there is significant heterogeneity, the treatment effect did not change with sensitivity testing. These results in particular are not different than the meta-analysis performed by Al-Halabi et. al. [13], which included four RCTs. The increase in LVEF is also consistent with a meta-analysis of observational studies [14].

The improvement in LVEF and 6MWT distance provides objective evidence for the benefit of AF ablation in patients with systolic HF. Whether this translates to a net benefit in the hard primary endpoints such as reduction in heart failure hospitalizations and mortality is not yet well-established. In our analysis, we noticed significant reduction in all-cause mortality in the AF ablation arm (OR 0.49; 95% CI: 0.31 – 0.77; P = 0.002). Despite this reduction, our confidence in this outcome is low since only the CASTLE AF trial [11] was designed to evaluate this outcome. HF-related hospitalizations were evaluated in the AATAC and CASTLE AF trials, and were significantly lower in the ablation arm (26.7% vs. 45.1%, OR 0.43; 95% CI: 0.29 – 0.64; P< 0.001). Nonetheless, lack of blinding may have led to potential bias in the outcome assessment.

The majority of patients in the trials had persistent AF (80.1%) and had NYHA functional class of II-III, thus limiting the ability to generalize the results. Namely, the results may not be extrapolated to patients with paroxysmal AF or those with asymptomatic (NYHA I) or severe (NYHA IV) functional classification. Regardless, our results show that in patients with persistent AF, where rate control is often committed, a rhythm control strategy using AF ablation as a tool might be beneficial.

Complications related to AF ablation although not common are also not rare. In the present meta-analysis, the overall complication rate was 33 (8.3%). About two-thirds of the complications were driven by stroke, cardiac tamponade, pericardial effusion, pulmonary venous stenosis and heart failure exacerbations. This rate of complications is higher than what is reported in a large cohort study (5.2%) [15] and a meta-analysis of 83,236 patients (2.9%) [16]. The higher rate of complications in our study may be explained by the structurally abnormal hearts in the population examined, and should be taken in consideration when referring such patients to AF ablation.

## 5. Conclusions

In patients with AF and coexisting HF, an ablation strategy results in improved LV function, functional capacity, and quality of life. This benefit might translate into improvement in hard outcomes, but more studies are needed to validate this idea. Overall, data from the present meta-analysis support the use of AF ablation in selected patients with HF.

## Figures and Tables

**Table 1:** Characteristics of patients included in the studies.

	Khan		MacDonald		Jones		Hunter		Di Biase		Prabhu		Marrouche	
	Ablation arm	AV ablation/ CRT	Ablation arm	Rate control	Ablation arm	Rate control	Ablation arm	Rate control	Ablation arm	Amiod-arone	Ablation arm	rate control	Ablation arm	Medical therapy
**Mean age (yrs)**	60 ± 8	61 ± 8	62.3 ± 6.7	64.4 ± 8.3	64 ± 10	62 ± 9	55 ± 12	60 ± 10	62 ± 10	60 ± 11	59 ± 11	62 ± 9.4	64	64

**Female gender**	5%	12%	23%	21%	19%	8%	4%	4%	25%	27%	6%	12%	13%	16%

**No. of patients**	41	40	22	19	26	26	26	24	102	101	33	33	179	184

**Follow up (months)**	6	6	9.7	6.9	12	12	12	6	24	24	6	6	37.6 ± 20.4	37.4 ± 17.7

**Persistent AF**	51%	46%	100%	100%	100%	100%	96%	88%	100%	100%	100%	100%	70%	65%

**NYHA class**	II & III	II & III	II & III	II & III	II & III	II & III	II & III	II & III	II & III	II & III	≥II	≥II	I-IV	I-IV

**ICMP**	N/A	N/A	50%	47%	38%	27%	23%	29%	62%	65%	0%	0%	40%	52%

**NICMP**	N/A	N/A	50%	53%	62%	73%	77%	71%	38%	35%	100%	100%	60%	48%

**LVEF **%	27 ± 8	29 ± 7	36.1 ± 11.9	42.9 ± 9.6	22 ± 8	25 ± 7	31.8 ± 7.7	33.7 ± 12.1	29 ± 5	30 ± 8	32 ± 9.4	34 ± 7.8	32.5	31.5

**LA diameter (mm)**	49 ± 5	47 ± 6	N/A	N/A	50 ± 6	47 ± 7	52 ± 11	50 ± 10	47 ± 4	48 ± 5	48 ± 6	47 ± 8	48	49.5

**6 min walk distance**	269 ± 54	281 ± 44	317.5 ± 125.8	351.8 ± 117.1	416 ± 78	411 ± 109	N/A	N/A	348 ± 111	350 ± 130	491 ± 147	489 ± 132	N/A	N/A

**Peak VO2**	N/A	N/A	N/A	N/A	16.3 ± 5.3	18.2 ± 4.8	N/A	NA	N/A	N/A	N/A	N/A	N/A	N/A

**Quality of life**	89 ± 12	89 ± 11	55.8 ± 19.8	59.2 ± 22.4	42 ± 23	49 ± 21	N/A	N/A	52 ± 24	50 ± 27	N/A	N/A	N/A	N/A

**Table 2:** Medications use after randomization.

	Khan		MacDonald		Jones		Hunter		Di Biase		Prabhu		Marrouche	
	Ablation arm	AV ablation/ CRT	Ablation arm	Rate control	Ablation arm	Rate control	Ablation arm	Rate control	Ablation arm	Amiodarone	Ablation arm	rate control	Ablation arm	Medical therapy
**Rate control **	NA	NA	*β*B ± digoxin	*β*B ± digoxin	*β*B ± digoxin	*β*B ± digoxin	*β*B	*β*B	*β*B ± digoxin	*β*B ± digoxin	*β*B	*β*B	*β*B ± digoxin	*β*B ± digoxin

**AAD**	5 patients received amiodarone, 4 received class III AAD and 1 received class IC AAD	14 patients received amiodarone and 1 received class III AAD	Oral amiodarone for 3 months in all patients	None	AAD stopped post ablation unless indicated by other reasons	None	AAD stopped post ablation unless indicated by other reasons	None	AAD allowed for 3 months after the first ablation	Amiodarone in all	9 patients received amiodarone and 3 received sotalol	None	45 patients received amiodarone, and 2 received sotalol	56 patients received amiodarone, and 6 received sotalol

**Anticoagulation**	Warfarin for at least 3 months, then at the discretion of the treating physician	NA	Warfarin	Warfarin	N/A	N/A	Warfarin	Warfarin	N/A	N/A	Per guidelines	Per guidelines	Warfarin for at least 6 months, then at the discretion of the treating physician	Per guidelines

**AAD**: antiarrhythmic drugs, **βB**: beta-blockers, and **NA**: not available.

**Table 3:** Risk of bias assessment table.

**Bias**	**Study**	**Judgement**	**Support for judgement**
Random sequence generation (selection bias)			

	Khan 2008	Low risk	Computer generated

	MacDonald 2011	Low risk	Computer generated

	Jones 2013	Low risk	Computer generated

	Hunter 2014	Low risk	Random number generator

	Di Biase 2016	Low risk	Computer generated

	Prabhu 2017	Low risk	Computer generated

	Marrouche 2018	Low risk	Computer generated

Allocation concealment (selection bias)			

	Khan 2008	Low risk	Computer generated randomization

	MacDonald 2011	Low risk	Computer generated randomization

	Jones 2013	Low risk	Computer generated randomization

	Hunter 2014	Low risk	Random number generator

	Di Biase 2016	Low risk	Computer generated randomization

	Prabhu 2017	Low risk	Computer generated randomization

	Marrouche 2018	Low risk	Computer generated randomization

Blinding of participants and personnel (performance bias)			

	Khan 2008	High risk	No blinding

	MacDonald 2011	High risk	No blinding

	Jones 2013	High risk	No blinding

	Hunter 2014	High risk	No blinding

	Di Biase 2016	High risk	No blinding

	Prabhu 2017	High risk	No blinding

	Marrouche 2018	High risk	No blinding

Blinding of outcome assessment (detection bias)			

	Khan 2008	High risk	No blinding

	MacDonald 2011	Moderate risk	Only scans analysis was blinded

	Jones 2013	Low risk	People conducting cardiopulmonary exercise test and imaging analysis were blinded

	Hunter 2014	Moderate risk	Only echocardiogram analysis was blinded

	Di Biase 2016	Moderate risk	Only echocardiogram analysis was blinded

	Prabhu 2017	High risk	No blinding

	Marrouche 2018	High risk	No blinding

Incomplete outcome data addressed (attrition bias)			

	Khan 2008	Low risk	No significant attrition

	MacDonald 2011	Low risk	No significant attrition

	Jones 2013	Low risk	No significant attrition

	Hunter 2014	Low risk	No significant attrition

	Di Biase 2016	Low risk	No significant attrition

	Prabhu 2017	Low risk	No significant attrition

	Marrouche 2018	Low risk	No significant attrition

Selective reporting (reporting bias)			

	Khan 2008	Low risk	

	MacDonald 2011	Low risk	

	Jones 2013	Low risk	

	Hunter 2014	Low risk	

	Di Biase 2016	Low risk	

	Prabhu 2017	Low risk	

	Marrouche 2018	Low risk	

**Table 4:** Intervention and follow up.

	Khan	MacDonald	Jones	Hunter	Di Biase	Prabhu	Marrouche
**Frequency of monitoring (months)**	3 & 6	3 & 6	3,6 & 12	1, 3 & 6	3, 6, 12 & 24	3 & 6	3, 6, 12, 24, 36, 48 & 60

**Method of assessing rhythm**	Loop recorder	24h holter monitor	48h holter monitor ± existing implantable devices	48h holter monitor	ECG, and existing implantable devices	24h holter monitor and ILR	Existing implantable devices

**Repeat procedure**	8 (19.5%)	6 (28.6%)	5 (19.2%)	14 (53.8%)	1.4 ± 0.6 per person	Repeat procedure was allowed (frequency not defined)	37 (24.5%)

**Crossover**	None	None	2	None	None	None	46

**Loss to follow-up**	None	3	None	1	None	1	33

**Ablation strategy**	PVI ± Linear lesions ± left atrial complex fractionated electrograms	PVI ± Linear lesions ± left atrial complex fractionated electrograms ± Cardioversion ± cavotricuspid isthmus ablation	PVI ± Linear lesions ± left atrial complex fractionated electrograms ± Cardioversion ± cavotricuspid isthmus ablation.	PVI ± Linear lesions ± left atrial complex fractionated electrograms ± Cavotricuspid isthmus ablation	PVI, and left atrial posterior wall isolation ± SVC isolation ± Linear lesions ± left atrial complex fractionated electrograms ± cardioversion	PVI, left posterior wall isolation ± cardioversion	PVI, Additional ablation lesions were made at the discretion of the operators

**PVI:** pulmonary veins isolation and **SVC:** superior vena cava.

**Table 5:** Complications related to catheter ablation.

	Khan (N = 41)	MacDonald (N = 21)	Jones (N = 25)	Hunter (N = 26)	Di Biase (N = 102)	Prabhu (N = 33)	Marrouch (N = 151)	Total (N=399)
**Death, N (**%**)**	0 (0.0%)	0 (0.0%)	0 (0.0%)	0 (0.0%)	0 (0.0%)	0 (0.0%)	0 (0.0%)	0 (0.0%)

**Stroke, N (**%**)**	0 (0.0%)	1 (4.8%)	0 (0.0%)	1 (3.8%)	0 (0.0%)	0 (0.0%)	0 (0.0%)	2 (0.5%)

**Cardiac tamponade, N (**%**)**	0 (0.0%)	2 (9.5%)	1 (4.0%)	1 (3.8%)	0 (0.0%)	0 (0.0%)	0 (0.0%)	4 (1.0%)

**Pericardial effusion, N (**%**)**	1 (2.4%)	0 (0.0%)	0 (0.0%)	0 (0.0%)	1 (1.0%)	0 (0.0%)	3 (2.0%)	5 (1.3%)

**Pulmonary vein stenosis, N (**%**)**	2 (4.9%)	0 (0.0%)	0 (0.0%)	0 (0.0%)	0 (0.0%)	0 (0.0%)	1 (0.7%)	3 (0.8%)

**Access site complications, N (**%**)**	3 (7.3%)	0 (0.0%)	1 (4.0%)	0 (0.0%)	2 (2.0%)	1 (3.0%)	6 (4.0%)	13 (3.3%)

**Heart failure exacerbation, N (**%**)**	1 (2.4%)	3 (14.3%)	1 (4.0%)	0 (0.0%)	0 (0.0%)	0 (0.0%)	1 (0.7%)	6 (1.5%)
